# Supplementary material for: Community Occupational Therapy for people with dementia and family carers (COTiD-UK) versus treatment as usual (Valuing Active Life in Dementia [VALID]) study: A single-blind, randomised controlled trial
Source: PLoS Med. 2021 Jan 4;18(1):e1003433. doi: 10.1371/journal.pmed.1003433 (PMC7781374; doi:10.1371/journal.pmed.1003433)
Supplement: S1 Statistical Analysis Plan — (PDF) [file pmed.1003433.s002.pdf]

VALID Trial

Statistical Analysis Plan

FINAL VERSION 1.0

20 MARCH 2018

1 Contents

2 Study Summary 3
3 List of Abbreviations 4
4 Introduction 5
4.1 Purpose of the Statistical Analysis Plan 5
4.2 Authorship 5
4.3 Organisation of Data and Analyses 5
5 Summary of Quantitative Trial Data 6
5.1 Observation times 6
5.2 Summary of Outcome Measures 6
5.2.1 Primary outcome 6
5.2.2 Secondary outcomes 6
5.3 Other available data 7
6 Analyses 8
6.1 Recruitment and Retention 8
6.2 Protocol Deviations 8
6.3 Description of Demographic Variables at Baseline 8
6.4 Drop-out 8
6.5 Primary Outcome Analysis 8
6.5.1 Model Checking 10
6.5.2 Missing Data 10
6.6 Secondary Outcome Analyses 10
6.7 Non-adherence 11
6.8 Reporting 11
7 References 12

2 Study Summary

Title: Valuing Active Life in Dementia (VALID): A randomised controlled trial of Community Occupational Therapy in Dementia (COTiD-UK).

Design: A multi-centre, pragmatic, single blind, two-arm randomised controlled trial in people with dementia and their family carers (with a person with dementia-carer pair termed a 'dyad'), to compare the COTID-UK intervention, offered in addition to treatment as usual (TAU), with treatment as usual alone (1).

Aims: To test the hypotheses that, when compared to TAU alone, COTiD-UK will:

- Significantly improve activities of daily living ability (e.g. self-care, leisure activities) in individuals with dementia.
- Significantly improve the quality of life of individuals with dementia and their family carers.
- Demonstrate cost effectiveness

Outcome measures: Primary outcome:

- Bristol Activities of Daily Living Scale (BADLS) (2)

Secondary outcomes:

- Mini Mental State Examination (MMSE) (4)
- Interview of deterioration in daily activities in dementia (IDDD) (5)
- Dementia Quality of Life Scale (DEMQOL) (6)
- Dementia Quality of Life Scale – proxy completed by carer
- Cornell Scale for Depression in Dementia (CSDD) (7)
- Serious Adverse events
- Sense of competence questionnaire (SCQ) (8)
- Hospital anxiety and depression scale (HADS) (9)
- Social contacts
- Social activities

Population: Individuals with dementia, living in the community, and their family carers.

Eligibility: Individuals with a diagnosis of dementia and living in the community (i.e. their own home - including sheltered accommodation) with a family carer who provides at least four hours of support per week. Individuals are not eligible to participate if they are currently in hospital, are currently participating in another intervention research study or do not have the capacity to consent.

3 List of Abbreviations

BADLS	Bristol Activities of Daily Living Scale
CACE	Complier average causal effect
COTiD-UK	Community Occupational Therapy in Dementia, UK version (developed in VALID work packages 1 and 2)
CSDD	Cornell Scale for Depression in Dementia
DEMQOL	Dementia Quality of Life Scale
Dyad	Patient with dementia and his/her family carer pairing
HADS	Hospital Anxiety and Depression Scale
IDDD	Interview of Deterioration in Daily activities in Dementia
MMSE	Mini Mental State Examination
SAP	Statistical analysis plan
SCQ	Sense of Competency Questionnaire
TAU	Treatment as usual
VALID	Valuing Active Life in Dementia

4 Introduction

4.1 Purpose of the Statistical Analysis Plan

This document contains details of the main quantitative, statistical, analyses for the VALID trial. These analyses shall be pre-specified in order that they are not influenced by the collected trial data after unmasking. The statistical analysis plan (SAP) does not preclude the undertaking of further, ad-hoc, analyses, although the results of any such further analyses should be interpreted carefully. Furthermore, the SAP does not prevent the adaptation of any part of the trial analysis, should situations arise in which such adaptation is deemed necessary. Any such adaptation shall be fully justified and transparent.

The SAP contains details of quantitative analyses only and does not describe any qualitative and/or economic analyses.

4.2 Authorship

The SAP has been written by Dr. Aidan O'Keeffe and Prof. Rumana Omar.

4.3 Organisation of Data and Analyses

Unmasking of collected data shall occur after the final dyad has completed follow-up, once all data have been entered onto the trial database and checked, the database locked for analysis and the SAP has been finalised and approved. The programs and code to be used for statistical analyses shall be prepared, where possible, prior to the unmasking of data. Two statisticians shall perform the analysis relating to the primary outcome independently, in order to ensure its accuracy.

Prior to performing analyses, basic checks shall be performed on the collected, masked, data to ensure accuracy. Each outcome (primary and secondary) variable and baseline demographic variable shall be checked for:

- missing values;
- values beyond an acceptable range;
- other inconsistencies.

If missing values or other inconsistencies are present, the corresponding data shall be checked with the aid of the researchers and, where necessary, either corrected or deemed to be missing. Any such changes made to the unmasked dataset shall be documented fully.

5 Summary of Quantitative Trial Data

5.1 Observation times

The times at which data are collected during the trial are as follows:

- Baseline (week 0);
- Week 12;
- Week 26;
- Week 52 (telephone follow-up – 75% of the sample);
- Week 78 (telephone follow-up – 25% of the sample).

The data recorded at the baseline, week 12 and week 26 time points shall constitute the full dataset, for the purpose of analysis of the primary outcome. At each time point beyond baseline, data shall be considered recorded at a given time point, provided that these data are collected from each dyad not more than two weeks before and up to 30 days after the corresponding time point. Any dyads from whom data are not collected within this time limit shall be considered missing at the affected follow-up times for the purpose of the primary statistical analysis. For the primary outcome, a supportive analysis will be performed that includes any data from dyads that were not recorded within the stated collection window for a given time point.

5.2 Summary of Outcome Measures

5.2.1 Primary outcome

The primary outcome measure is the BADLS measured at week 26. The sample size for the trial has been calculated to have 90% power to detect an effect size of 0.35 SMD using a two-tailed, two-sample t-test (significance level of 0.05) comparing mean BADLS at week 26 between TAU and COTiD-UK groups.

5.2.2 Secondary outcomes

1. BADLS measured at other time points;
2. Mini Mental State Examination (MMSE);
3. Interview of Deterioration in Daily activities in Dementia (IDDD);
4. Dementia quality of life measure (DEMQOL) (from patient with dementia and by proxy from carer);
5. Cornell Scale for Depression in Dementia (CSDD);
6. Number and nature of serious adverse events;
7. Sense of Competence Questionnaire (SCQ);
8. Hospital Anxiety and Depression Scale (HADS), to be analysed separately as HADS anxiety and HADS depression scores;
9. Social contacts;
10. Social activities;

Furthermore, a collection of basic demographic information shall be made at baseline, for both the individual with dementia and his/her family carer, prior the collection of all other data. This information shall include:

- Date of birth (age);
- Gender;
- Marital status;
- Family carer / person with dementia relationship
- Ethnicity;
- Level of education.

Table 1 provides a summary of the times at which outcomes are collected.

Table 1: Table showing the outcome measures, the time points at which outcomes are collected and from whom the data are recorded.

**Outcome Measure**	**Data collected from**		**Data collected refer to**		**Time point**				
	**Person with dementia**	**Family carer**	**Person with dementia**	**Family carer**	**Baseline interview**	**Week 12 interview**	**Week 26 interview**	**Week 52 telephone interview**	**Week 78 telephone interview**
BADLS	No	Yes	Yes	No	Yes	Yes	Yes	Yes	Yes
MMSE	Yes	No	Yes	No	Yes	Yes	Yes	No	No
IDDD	No	Yes	Yes	No	Yes	Yes	Yes	No	No
DEMQOL Proxy	No	Yes	Yes	No	Yes	Yes	Yes	No	No
DEMQOL	Yes	No	Yes	No	Yes	Yes	Yes	No	No
CSDD	Yes	No	Yes	No	Yes	Yes	Yes	No	No
SCQ	No	Yes	No	Yes	Yes	Yes	Yes	No	No
HADS	No	Yes	No	Yes	Yes	Yes	Yes	Yes	Yes
Serious Adverse Events	Yes	Yes	Yes	Yes	Yes	Yes	Yes	Yes	Yes
Social contacts per week	No	Yes	Yes	Yes	Yes	Yes	Yes	Yes	Yes
Social activities per week	No	Yes	Yes	Yes	Yes	Yes	Yes	Yes	Yes

5.3 Other available data

Other available data may be summarised as:

- Centre identifier;
- Identifier of occupational therapist (COTiD-UK arm only);
- Information relating to TAU (TAU arm only).
- Reasons for withdrawal or loss to follow-up (if supplied).
- Dates of assessments.

6 Analyses

6.1 Recruitment and Retention

A CONSORT diagram shall be presented to provide a detailed description of dyad numbers at each time point during the trial. In addition, a table summarising the numbers of drop-outs at each stage of the trial and reasons for drop-out (if given) shall be presented.

6.2 Protocol Deviations

Protocol deviations that could impact on the results of the analyses shall be described. All dyads who withdraw consent shall be excluded from the analyses from the point of withdrawal, although any data collected from such dyads prior to the point of withdrawal shall be included unless the dyad specifies otherwise.

6.3 Description of Demographic Variables at Baseline

The demographic information collected at baseline shall be presented in a table, separately for individuals with dementia and their family carers and separated by trial arm. Categorical variables shall be reported as raw numbers and percentages. Reports of continuous variables shall include mean, median and standard deviation, to two decimal places.

6.4 Drop-out

A descriptive comparison of drop-out rates between trial arms shall be undertaken.

6.5 Primary Outcome Analysis

The primary outcome is the BADLS score measured at week 26, to be compared between the COTiD-UK and TAU groups. A random effects linear regression model shall be fitted, accounting for the clustering of dyads by occupational therapist in the COTiD-UK arm. The model shall include an adjustment for BADLS measured at baseline and a term to account for treatment centre. For each dyad, the mean BADLS outcome will also be calculated by computing the mean of the responses to the individual questions on the BADLS questionnaire, at a given time point, using only those answers that were not marked as "Not Applicable" or that were not missing.

Define (where the i subscript denotes the ith dyad, the j subscript denotes the jth occupational therapist and the k subscript denotes the kth treatment centre.):

- Y_{ijk} = BADLS score at week 26;
- x_{ijk} = BADLS score at baseline;
- t_{ijk} = Intervention indicator (t_{ijk} = 0 if dyad is in the TAU arm and t_{ijk} = 1 if dyad is in the COTiD-UK arm);
- $U_j \sim N(0, \tau^2)$ = Occupational therapist-level random effect;
- $\varepsilon_{ijk} \sim N(0, \sigma_1^2)$ = Normally distributed error term (TAU arm);
- $\omega_{ijk} \sim N(0, \sigma_2^2)$ = Normally distributed error term (COTiD-UK arm).

- α_k = Fixed effect for the kth treatment centre

A subscript ijk implies that the ith dyad is treated by the jth occupational therapist at the kth treatment centre. For dyads in the TAU group, the j subscript may be removed without loss of generality.

Then, the model for the primary outcome is given by:

$$Y_{ijk} = \mu + \alpha_k + \beta_1 x_{ijk} + \beta_2 t_{ijk} + U_j t_{ijk} + \omega_{ijk} t_{ijk} + \varepsilon_{ijk}(1 - t_{ijk}).$$

Here, $(\mu, \alpha_k, \beta_1, \beta_2, \tau, \sigma_1, \sigma_2)^{\mathrm{T}}$ denotes the collection of parameters to be estimated when fitting the model.

We note that the trial design contains partial nesting, in that the dyads are clustered by the occupational therapist who administers the intervention in the COTiD-UK arm, but there is no such clustering in the TAU arm. This clustering is accounted-for in the model through the inclusion of the occupational therapist-level random effects U_j. By definition, $U_j \equiv 0$ for dyads in the TAU arm. In addition, we allow the patient-level variance of the outcome, Y_{ijk}, to differ between the COTiD-UK arm and the TAU arm, in that the error terms for these arms, ω_{ijk} and ε_{ijk} respectively have different variance parameters.

A further model that assumes homogenous patient-level variances in both arms (i.e. where $\sigma_1^2 = \sigma_2^2$) shall be fitted and compared to the model with heterogeneous patient-level variances. If no significant differences are observed between the fits of these two models, then we shall consider reporting the second homogenous variance model as the final model for the primary outcome.

The primary outcome models shall be fitted to the observed outcomes variables, initially without imputation of missing data. A P-value pertaining to the hypothesis test of

$$H_0: \beta_2 = 0 \quad \text{versus} \quad H_1: \beta_2 \neq 0$$

shall be reported. A P-value ≥ 0.01 shall be reported to two decimal places; a P-value in the range 0.001-0.01 shall be reported to three decimal places and a P-value less than 0.001 shall be reported as ‘P-value < 0.001’.

6.5.1 Model Checking

The model for the primary outcome analysis includes an assumption that the primary outcomes, Y_{ijk}, are normally distributed. The normality of the primary outcome variable shall be assessed through the construction of appropriate histograms and normal quantile-quantile plots. If such plots suggest that the primary outcome variable is not normally distributed, then appropriate transformations of the primary outcome variable shall be considered.

The normality of the estimated patient-level error terms (ω_{ijk} and ε_{ijk}) shall be assessed using normal quantile-quantile plots. The homoscedasticity of the same estimated error terms shall be assessed using a scatter plot of the estimated error terms. Possible influential observations and outliers shall be identified. Sensitivity to such influential observations and/or outliers (if present) shall be considered.

6.5.2 Missing Data

Where a total score is missing owing to one or more items not having been completed on a given questionnaire, guidance relating to the calculation of total scores from the literature/score instructions, if any, will be followed. Statistical approaches to account for missing data will apply only to BADLS scores.

Bias due to missing data will be investigated by comparing the baseline characteristics of participants with and without missing values. Depending on the extent of missingness, the predictors of missing values will be identified using logistic regression. The primary analysis will be adjusted for those predictors of missing values which are related to missingness. We shall perform a sensitivity analysis for missing data in our primary outcome model. A multiple imputation approach may be undertaken (10), whereby a model to predict missing BADLS at Week 26 shall be constructed using appropriate explanatory variables. The variables that we shall consider are: the baseline BADLS score and the participant demographics, also recorded at baseline. Other variables thought to be related to missingness (e.g. BADLS at Week 12) may also be included. As a general rule, we shall consider imputed sets of outcome data equal to the percentage of missing data and imputation shall be performed with adjustment for treatment arm. The number of imputations carried out may be changed according to the computation time and Monte-Carlo error estimated from the imputation process. Initially, the multiple imputation process assumes that outcomes are ‘missing at random’.

6.6 Secondary Analyses

Part of the secondary analyses shall consider other models and tests pertaining to the primary outcome (BADLS). Within the COTiD-UK group, the effect of the experience of the individual occupational therapist shall be assessed. For this analysis, we shall consider the length of time for which the occupational therapist has been delivering the COTiD-UK intervention and/or the number of COTiD-UK sessions delivered by the occupational therapist as explanatory variables in a random effects model with BADLS as the outcome. In addition, the contribution of occupational therapy to TAU in the TAU arm shall be used as an explanatory variable in a random effects model with BADLS as the outcome variable in the TAU arm. This shall be done to explore whether or not there exists any association between the occupational therapy contribution to treatment and change in BADLS.

The analyses of secondary outcomes shall be performed using mixed effects regression models, with a normal distribution assumed for continuous outcomes (or a suitable transform of these outcomes) and appropriate mixed effects generalised linear models for non-continuous outcomes (e.g. number of social contacts). The mixed effects models will take a similar form to that for the BADLS primary outcome analysis. Model parameter estimates together with appropriate 95% confidence intervals shall be reported. These analyses shall be considered exploratory and secondary outcomes shall be analysed using available data only with imputation for missing outcomes not performed.

The repeated measurements of outcome variables at baseline, Week 12, Week 26, Week 52 and Week 78, (including BADLS) shall be considered and mixed effects longitudinal regression models that account for patient clustering shall be fitted using longitudinal outcomes.

In addition, joint models for the time until death/care home admission and the longitudinal BADLS scores maybe fitted if appropriate (e.g. if death/care home admission rates differ substantially between the arms of the trial).

The number, nature and severity of serious adverse events (if any) shall be reported at each time point, stratified by trial arm.

The components of usual care will be presented descriptively.

6.7 Non-adherence

The sample size calculation for the trial was adjusted for an estimated 5% rate of non-adherence in the COTiD-UK arm. Non-adherence within the COTiD-UK arm shall be examined descriptively. If appropriate, we shall consider the use of a complier average causal effect (CACE) of the COTiD-UK intervention as part of a sensitivity analysis with respect to non-compliance. The CACE is defined as:

$$CACE = \frac{E(Y_i|G_i = 1) - E(Y_i|G_i = 0)}{P(T_i = 1|G_i = 1) - P(T_i = 1|G_i = 0)}$$

where:

- Y_i = outcome variable;
- T_i = treatment indicator (= 1 if dyad actually takes part in COTiD-UK, 0 otherwise);
- G_i = randomisation indicator (= 1 if dyad is randomised to the COTiD-UK group, 0 otherwise).

6.8 Reporting

Analyses shall be reported with regard to the CONSORT checklist (11) and with any particular requirements of academic journals to which the results of analyses are submitted.

7 References

1. Wenborn J, Hynes S, Moniz-Cook E, Mountain G, Poland F, King M, Omar R, Morris S, Vernooij-Dassen M, Challis D, Michie S, Russell I, Sackley C, Graff M, O'Keeffe A, Crellin N, Orrell M. Community occupational therapy for people with dementia and family carers (COTiD-UK) versus treatment as usual (Valuing Active Life in Dementia [VALID] programme): study protocol for a randomised controlled trial. Trials. 2016; 17:65

2. Bucks RS, Ashworth DL, Wilcock GK, Siegfried K. Assessment of Activities of Daily Living in Dementia: Development of the Bristol Activities of Daily Living Scale. Age Ageing. 1996 Jan 3;25(2):113–20.

3. J.K. Beecham, M.R.J. Knapp. Costing psychiatric interventions. Measuring Mental Health Needs. London: Gaskell; 1992.

4. Folstein MF, Folstein SE, McHugh PR. 'Mini-mental state'. A practical method for grading the cognitive state of patients for the clinician. J Psychiatr Res. 1975 Nov;12(3):189–98.

5. S. Teunisse, M.M. Derix, H. van Cravel. Assessing the severity of dementia. Archives of Neurology. 48(3):274–7.

6. S.C. Smith, D.L. Lamping, S. Banerjee, R. Harwood, B. Foley, P. Smith, et al. Measurement of health-related quality of life for people with dementia: development of a new instrument (DEMQOL) and an evaluation of current methodology. Health Technology Assessment. 2005;9:1–93.

7. Alexopoulos GS, Abrams RC, Young RC, Shamoian CA. Cornell Scale for Depression in Dementia. Biol Psychiatry. 1988 Feb 1;23(3):271–84.

8. Vernooij-Dassen, M.J.F.J., Persoon J.M.G., Felling A.J.A. Predictors of Sense of competence in caregivers of demented persons. Social Science and Medicine. 43:41–9.

9. Zigmond AS, Snaith RP. The hospital anxiety and depression scale. Acta Psychiatr Scand. 1983 Jun;67(6):361–70.

10. James R. Carpenter, Michael G. Kenward. Multiple Imputation and its Application. Wiley; 2013.

11. Schulz KF, Altman DG, Moher D., CONSORT 2010 Statement: updated guidelines for reporting parallel group randomised. Trials. 2010 Mar 24;11(1):32.
