# Supplementary material for: Community Occupational Therapy for people with dementia and family carers (COTiD-UK) versus treatment as usual (Valuing Active Life in Dementia [VALID]) study: A single-blind, randomised controlled trial
Source: PLoS Med. 2021 Jan 4;18(1):e1003433. doi: 10.1371/journal.pmed.1003433 (PMC7781374; doi:10.1371/journal.pmed.1003433)
Supplement: Attachment — Submitted filename: PLOS Medicine Authors response UPDATED 060720.docx [file pmed.1003433.s003.docx]

**PLOS Medicine – D-20-00486R1**

**Community Occupational Therapy for people with dementia and family carers (COTiD-UK) versus treatment as usual (Valuing Active Life in Dementia [VALID]) study: a single-blind, randomised controlled trial**

| **Editorial / Reviewer comments:** | **Authors’ response** | **Page / line** |
| --- | --- | --- |
| To enhance the reproducibility of your results, we recommend that you deposit your laboratory protocols in protocols.io, where a protocol can be assigned its own identifier (DOI) such that it can be cited independently in the future. | I have looked at this website as suggested but question whether it is relevant with its focus being the basic sciences and as you describe it, being a repository for laboratory protocols as our study does not involve any laboratory specimens or activity. Our trial protocol is published in Trials as Open Access and so already has a DOI as follows below (see reference number 13 in manuscript), so might this be included as a link in the text?  Wenborn J, Hynes S, Moniz-Cook E, Mountain G, Poland F, King M, et al. Community Occupational Therapy for people with dementia and family carers (COTiD-UK) versus treatment as usual (Valuing Active Life in Dementia [VALID] study): study protocol for a randomised controlled trial. Trials 2016;17(65). doi:10.1186/s13063-015-1150-y. | N/A |
| Please ensure that the paper adheres to the PLOS Data Availability Policy (see link…. ), which requires that all data underlying the study's findings be provided in a repository or as Supporting Information. | Our Trial Statistician has formatted the trial dataset in readiness to upload to the UCL Data Repository prior to publication, at which point the URL will be provided.  Please note that in preparing the dataset an inconsistency in reporting the number of occupational therapists delivering the intervention was noted and this has now been corrected with an explanation in the manuscript. Subsequently the analysis was rerun and the slightly different values amended throughout. | 17, 391-393 |
| **Requests from the editors:** | | |
| At this stage, we'd appreciate it if you could structure your abstract using the PLOS Medicine headings (Background, Methods and Findings, Conclusions). The Methods and Findings sections are one combined sub-section, “Methods and findings”. Please also make sure each subsection is written in complete sentence text (ie not sentence fragments). | Our apology for the oversight. The Abstract has been amended to follow the required format. | 4-5 |
| In the last sentence of the Abstract Methods and Findings section, please summarise some of the key limitation(s) of the study's methodology. | A summary of the key limitations has been added at the end of the Methods and Findings section of the Abstract. | 5 |
| At this stage, we ask that you include a short, non-technical Author Summary of your research to make findings accessible to a wide audience that includes both scientists and non-scientists. The Author Summary should immediately follow the Abstract in your revised manuscript. This text is subject to editorial change and should be distinct from the scientific abstract. | An Author Summary has been inserted following the Abstract. | 6-7 |
| In some places in the main text, the writeup involves sentence fragments rather than complete sentences (eg, Methods/Study Design section). We'd suggest rewriting these sections so they are in complete sentences. | The manuscript has been edited as requested. | eg:  9, 191-193 |
| The completed CONSORT flowchart and checklist have been included with the paper (as figure/supporting information), which is great, we would just suggest also adding a short statement to the Methods section of the paper that the CONSORT guideline was used to guide reporting of the paper (and then to call out/reference the CONSORT checklist supporting information file). | We have amended the text to include a reference to the CONSORT Statement, Flowchart and Checklist – and uploaded a revised Checklist with the updated page numbers indicated. | 9, 185-187 |
| **Reviewer 1** |  |  |
| This is a very well written and well conducted study with important findings. I was very interested to read the results - Fidelity and the outcome measures used appear to be related to the lack of benefit detected. I suggest only two minor revisions: | Thank you for your time spent reviewing our manuscript and for your very positive comments. |  |
| 1) Please describe early in the paper which aspect of ADL the Bristol Measure uses (eg IADL, BADL, both, performance of, participation in etc....) as I and other readers will be less familiar with the Bristol measure | Clarification that the BADLS measures performance of personal care and instrumental ADL has been inserted in   1) the study Hypothesis paragraph 2) the outcome measures section. | 9, 179-184  14, 297-299 |
| 2) Can you report on how many consultations the intervention group received (will help with understanding fidelity) | The description of the COTiD-UK intervention in the Procedure / Interventions section has been amended to clarify the flexible delivery of the ten hours of community occupational therapy over ten weeks, rather than stipulating a set number of sessions.  Also, in response to Reviewer 3, the process and outcome of our adaptation of the Dutch intervention to develop the COTiD-UK version is now described in more detail in the Introduction section and as such also clarifies the more flexible nature of the intervention content and delivery. | 11-12,  246-250  8, 167-175 |
| **Reviewer 2:** | | |
| Thanks for the opportunity to review your manuscript. My role is as a statistical reviewer and so my comments focus on the study design, data, and analysis of the study (and the presentation of these). I have put general comments, and followed these with specific queries with a page/line reference.  The methods section mentions a prespecified plan - is this available? It would be very helpful when reviewing the statistical aspects of the manuscript, I think many of my queries below could be resolved with additional information from a SAP. | Thank you for your time spent in reviewing our manuscript and for your very useful comments.  Yes, there was a prespecified Statistical Analysis Plan (SAP) and we will upload this alongside the revised manuscript. |  |
| The study used a data monitoring and ethics committee to oversee trial conduct and safety (p13, L296), and has also been listed as being masked to the participants allocation. For the DMEC, does the masking refer to access to individual patient data or did the committee only have access to blinded monitoring reports throughout the study? | We have now explained this in the revised manuscript:  “This committee received Open and Closed reports with the latter remaining confidential to the DMEC members and Trial Manager and was NOT available to the Trial Statistician who attended a portion of each meeting. Baseline, reason for withdrawal, and serious adverse event data were provided by group allocated in the Closed report. All data in both reports were anonymised, and no outcome data were included.” | 16, 354-360 |
| P6, L140. It wasn't clear to me when I read this paragraph that BADLS was the main outcome of the study and that QoL and the other measures were secondary. | This sentence has been reworded as follows to improve the clarity as requested, and to incorporate Reviewer 1’s query regarding the aspect of ADL measured by the BADLS:  “Our aim was to estimate the clinical and cost effectiveness of COTiD-UK relative to TAU. We hypothesised that, in comparison with TAU, COTiD-UK would improve personal care and instrumental ADL performance in people with mild to moderate dementia measured using the Bristol Activities of Daily Living Scale (BADLS) score at 26 weeks as our primary outcome, as well as sense of competence and mood in family carers, and cognition, mood and quality of life for people with dementia and their family carers as secondary outcomes.” | 9, 179-184 |
| P7, L152. Was the rationale for excluding patients with more severe dementia because of issues around consent? | The rationale for excluding people with more severe dementia was due to the dyadic nature of the intervention that is specifically designed for people with mild to moderate dementia and their family carer to work in partnership together with the occupational therapist. It therefore requires the person with dementia to be able to participate accordingly, ie: to engage in the narrative interview and then set / enact personally meaningful goals along with their carer, and thus would not be suitable for those with more severe dementia.    The target population for the intervention and the study has been made clearer in the introduction, the hypothesis and the description of the intervention. | 8, 155-157; 9, 179-184;  11, 246-260 |
| P8, L182. What size block was used? Were study staff blinded to the block size? | The block sizes used were 2, 4, 7 and 14, and all study staff were masked to this.  This information has been inserted to the text. | 11, 229-230 |
| P10, L243. To clarify, was the follow-up at 52 weeks ceased once 77% of participants contacted and interviewed? How were the 40% at 78 weeks selected for follow-up? | This paragraph has been reworded as follows to improve the clarity as requested:  “We collected the complete dataset through interviews in participants’ homes at baseline, 12 and 26 weeks. We collected a reduced dataset from the carer by telephone at 52 and 78 weeks after randomisation. We had originally planned to collect these data for all pairs at 52 weeks and for the first 40% of pairs recruited to the trial at 78 weeks. However, due to delays in recruiting the research sites and then the pairs, alongside the need to complete the trial on time as agreed with the study funder, in practice we were able to follow up the first 77% of pairs that had been recruited to the trial at the 52 weeks timepoint (n = 368 pairs), and we met our original target of following up the first 40% of pairs who had been recruited to the trial at the 78 weeks timepoint (n = 192 pairs).” | 13, 288-295 |
| P12, L266. What criteria was used to decide if this is a clinically significant change? Was there consumer input into this criteria? | There was no consumer input to agreeing this criteria. More detail is now included in the Sample size section.  “The effect size of 0.35 was determined using the clinical expertise of the applicant group, based on the DOMINO group consensus advice regarding the minimum clinically important difference (MCID) using the BADLS. As such we took a more conservative approach than the findings of Graff et al. who demonstrated an effect size of 1.25 albeit in a single site trial with the intervention delivered by two therapists and using a different ADL measure.” | 14-15,  317-323 |
| P12, L270. Was the main analysis originalyl planned to be ITT given an adjustment for non-adherence is included? Was the allocation ratio adjusted from 1:1 to account for the 5% non-adherence in the intervention group? | The main analysis is by intention-to-treat, as planned. As we note in the ‘Statistical Methods‘ section some planned supportive analyses were undertaken, including a CACE analysis to account for non-adherence in the intervention group.  The allocation ratio for the sample size was adjusted from 1:1 to account for clustering by occupational therapist in the intervention group. We have re-worded the section on ‘Sample size’ to clarify this. This now states:  “The total sample size (for both groups) was inflated to allow for 15% attrition and 5% non-adherence at week 26. Additionally, we increased the size of the intervention group to account for within-therapist clustering, assuming an intraclass correlation coefficient (ICC) of 0.015 and an average of 10 pairs per therapist. In total, we planned to recruit 256 pairs to the intervention group and 224 pairs to the TAU group.” | 15,325-329 |
| P12, L275. Was the site effect included as a random intercept or a fixed effect? | The site effect was fixed and we have clarified this in the revised version of the manuscript. | 15, 333 |
| P13, L302. Was the data from the internal pilot blinded or unblinded? | Some additional explanation has been included in the revised manuscript:  “The clinical researcher responsible for training and supervising the occupational therapists delivering the intervention was unmasked to allocation and so reviewed the serious adverse events reported to ensure participant safety, and the COTiD-UK Checklist data to check accuracy and to monitor intervention fidelity, whilst all other data were reviewed by masked team members No individual participant data were presented to the PSC.” | 16, 355-360 |
| P15, L347. The use of these additional variables here wasn't clear (and might be cleared up if the SAP is available) to me. Was the idea to have a main analysis robust to MAR using the mixed-effect model repeated-measures (MRMM), and then extend this to include other factors associated with missingness? | The SAP has been submitted with the revised version of the manuscript. The use of additional variables is not related to repeated measures. These variables were included in the analysis model to preserve the MAR assumption. The primary outcome is BADLS at 26 weeks and thus the primary analysis for this study is not a repeated measures model. As stated in the SAP, baseline demographic variables were tested for association with missingness of BADLS at Week 26. Variables found to have a significant association with missingness (ethnicity of person with dementia and marital status of the family carer) were then included in an adjusted model for the primary outcome (BADLS at week 26) as part of the supportive analysis, with an implicit MAR assumption. | N/A |
| P25, Fig 2. I'd consider including the modelled estimates of BADLS from the linear mixed models (marginal means) in addition to the summary measures, as these will include the adjustment for data that is missing at random used to test for differences between arms. | Figure 2 is a descriptive plot designed to show raw data summaries prior to any modelling. Marginal displays of means at each time point by group would not be appropriate owing to the use of the fixed site effect. | N/A |
| P31, Tab 3. Which part of the table does the footnote apply to | Thank you for highlighting this error which has been corrected in our revised manuscript. This table shows raw summaries of outcomes with no adjustments. | 30 |
| **Reviewer 3:** | | |
| This is an interesting and important paper. The methodology of the research is well described, strong and accurate. Although the results of the research are disappointing, publishing papers on interventions that did not yield the expected results is just as important as publishing about successful interventions. Moreover, possible explanations for the lack of effects are also part of the paper. The paper is well written and clear.  I have two minor comments: | Thank you for your time spent reviewing our manuscript and for your very positive comments. |  |
| In the introduction, the authors mention that the COTiD intervention has been adapted to make it more suitable to the UK. There however is no mention of what these adaptations are. This has probably been described in the protocol paper the authors refer to, but in view of trying to understand why the intervention does not have the intended effect, I think there are good reasons for (re)listing these adaptations here, and to assess whether these adaptations may have had an impact on the results and be part of the explanation of why the results in the Netherlands were different. | Further description of how COTiD was adapted to produce COTiD-UK has been added to the Introduction section. | 8, 167-176 |
| At the end of the paper, when comparing the study to similar studies in other countries, the authors state that usual care in the UK often encompasses occupational therapy. Have the participants been asked if and how much OT they received before the intervention? If so, should the number of hours receiving occupational therapy not be mentioned in table 1? | Thank you, this is an interesting question. Yes, participants were asked as part of the Client Services Resource Inventory (CSRI) if they had previously received any occupational therapy input. However these data were part of the economic evaluation and will thus be reported separately – that paper is currently in preparation.  It may be worth noting that if participants mentioned occupational therapy input when completing the CSRI at follow-up we had a system in place at each site for an unmasked researcher to check if this intervention had been provided as part of the COTiD-UK trial or was TAU. As we note in the final paragraph of the Results section we found no evidence that TAU occupational therapy had been provided by a COTiD-UK trained therapist so there was no contamination between the groups. |  |
