# Supplementary material for: Community Occupational Therapy for people with dementia and family carers (COTiD-UK) versus treatment as usual (Valuing Active Life in Dementia [VALID]) study: A single-blind, randomised controlled trial
Source: PLoS Med. 2021 Jan 4;18(1):e1003433. doi: 10.1371/journal.pmed.1003433 (PMC7781374; doi:10.1371/journal.pmed.1003433)
Supplement: Attachment — Submitted filename: PMEDICINE-D-20-00486R3 author response.docx [file pmed.1003433.s004.docx]

**PMEDICINE-D-20-00486R3**

**Community Occupational Therapy for people with dementia and family carers (COTiD-UK) versus treatment as usual (Valuing Active Life in Dementia [VALID]) study: a single-blind, randomised controlled trial**

| **Requests from Production Team (the page / line numbers refer to the clean version)** | | **Page / line** |
| --- | --- | --- |
| 1. Thank you for clarifying the data availability. Please let us know when the entry has gone live, as we cannot accept the paper until then. | I will confirm its publication as soon as possible. |  |
| 2. Please ensure that all authors have at least one affiliation listed on the title page and that all listed affiliations are attributed to at least one author. Affiliations should be listed in ascending numerical order in the affiliation list and should match what is provided in the Authors Information in the submission form. Affiliations should include an institution, a city, and a country (no postcode). | The Title page and the authors’ details provided in the submission form have been amended. My apologies for using the incorrect format previously. | 1 / Submission form |
| 3. Please remove the funding information from the Acknowledgments section of your manuscript file. The published version is pulled automatically from the “Financial Disclosure” field in our submission form and must not be duplicated. Please therefore take this opportunity to check that the information in this field is correct and completed in full. | The funding information has been removed from the Acknowledgements section of the manuscript. | 22 / Submission |
| 4. Please ensure that all Supporting Information files use the following format verbatim throughout the manuscript (including legends and all in-text citations): S1 Fig, S1 Table, S1 Text, S1 Data, etc. Each file type should start at 1 and increase numerically.  Please note, however, that the file names themselves must use an underscore rather than a space, i.e. S1_Fig.tif, S1_Table.xlsx, S1_Text.doc, S1_Data.xlsx, S1_CONSORT_Checklist.docx, S1_Protocol.pdf, etc. | There are two Supporting Information files. These are referred to in the manuscript text as S1 CONSORT Checklist (lines 147-148) and S1 Statistical Analysis Plan (line 288).  The uploaded files use the required format: S1_CONSORT_Checklist.docx, and S1_Statistical_ Analysis_Plan.pdf. | 7, 13  Submission form |
| 5. Please ensure that each supporting information file has a legend at the end of the manuscript file, after the Reference list. | A legend for the two Supporting Information files has been inserted after the Reference List. | 25 |
| 6. Please note that your submission must refer to specific files using our naming convention (i.e. S1 Fig, S1 Table, S1 Text, S1 Data, S1 Protocol, S1 CONSORT Checklist, etc.) | This has been done. | 8 |
